# Myogenetic Oligodeoxynucleotide (myoDN) Recovers the Differentiation of Skeletal Muscle Myoblasts Deteriorated by Diabetes Mellitus

**DOI:** 10.3389/fphys.2021.679152

**Published:** 2021-05-24

**Authors:** Shunichi Nakamura, Shinichi Yonekura, Takeshi Shimosato, Tomohide Takaya

**Affiliations:** ^1^Department of Agriculture, Graduate School of Science and Technology, Shinshu University, Nagano, Japan; ^2^Department of Agricultural and Life Sciences, Faculty of Agriculture, Shinshu University, Nagano, Japan; ^3^Department of Biomolecular Innovation, Institute for Biomedical Sciences, Shinshu University, Nagano, Japan

**Keywords:** myogenetic oligodeoxynucleotide, skeletal muscle, myoblast, myogenic differentiation, diabetes mellitus

## Abstract

Skeletal muscle wasting in patients with diabetes mellitus (DM) is a complication of decreased muscle mass and strength, and is a serious risk factor that may result in mortality. Deteriorated differentiation of muscle precursor cells, called myoblasts, in DM patients is considered to be one of the causes of muscle wasting. We recently developed myogenetic oligodeoxynucleotides (myoDNs), which are 18-base single-strand DNAs that promote myoblast differentiation by targeting nucleolin. Herein, we report the applicability of a myoDN, iSN04, to myoblasts isolated from patients with type 1 and type 2 DM. Myogenesis of DM myoblasts was exacerbated concordantly with a delayed shift of myogenic transcription and induction of interleukins. Analogous phenotypes were reproduced in healthy myoblasts cultured with excessive glucose or palmitic acid, mimicking hyperglycemia or hyperlipidemia. iSN04 treatment recovered the deteriorated differentiation of plural DM myoblasts by downregulating myostatin and interleukin-8 (IL-8). iSN04 also ameliorated the impaired myogenic differentiation induced by glucose or palmitic acid. These results demonstrate that myoDNs can directly facilitate myoblast differentiation in DM patients, making them novel candidates for nucleic acid drugs to treat muscle wasting in patients with DM.

## Introduction

The skeletal muscle is the largest organ (30–40% of total body weight) in human (Janssen et al., 2000), the primary thermogenic tissue producing heat by muscular contraction (Rowland et al., 2015), the main energy storage holding 500 g of glycogen (Jensen et al., 2011), and carries 80% of insulin-responsive glucose uptake (Periasamy et al., 2017). Therefore, a decrease in skeletal muscle mass disturbs systemic homeostasis and increases mortality in chronic diseases. Skeletal muscle wasting is present in around 30% of patients with heart failure and is associated with poor prognosis, probably in part, by increased inflammation and oxidative stress (Bielecka-Dabrowa et al., 2020). Loss of skeletal muscle mass is present in up to 50% of cancer patients and is a predictor for lower survival during chemotherapy as it increases treatment toxicity (Gibson et al., 2015). Low skeletal muscle mass is also observed in patients with diabetes mellitus (DM). Protein synthesis in skeletal muscle is decreased by type 1 DM (T1DM) due to loss of insulin signaling, and protein degradation is enhanced with myostatin-mediated autophagy in type 2 DM (T2DM) (Sala and Zorzano, 2015). Particularly, an age-adjusted study reported that muscle wasting is an independent risk for all-cause mortality in patients with T2DM (Miyake et al., 2019). Prevention of DM-associated muscle loss is an important subject but there is no efficacious drugs, because it is still a complicated pathology and underlying mechanism has not been fully understood.

The skeletal muscle is composed of a large number of myofibers, which are multinuclear fused myocytes. Each myofiber has dozens of stem cells, termed satellite cells, between the basal lamina and plasma membrane of the fibers. During myogenesis, satellite cells are activated to myogenic precursor cells, called myoblasts. Following this, myoblasts differentiate into mononuclear myocytes expressing myosin heavy chain (MHC), and mutually fused to form multinuclear myotubes (Dumont et al., 2015). Importantly, the satellite cell- and myoblast-driven myogenesis is impaired by DM, which is considered a predisposing factor for skeletal muscle loss. Both T1DM and T2DM deteriorate the functions of satellite cells and myoblasts owing to oxidative stress, chronic inflammation, extracellular matrix defects, and transcriptional disorders (D’Souza et al., 2013; Teng and Huang, 2019). In patients with T1DM, the number of satellite cells decreases with the upregulation of the Notch ligand DLL1 (D’Souza et al., 2016). Myoblasts isolated from patients with T2DM show impaired myogenic differentiation with lower miR-23b/27b levels (Henriksen et al., 2017) and autophagy dysregulation (Henriksen et al., 2019). Even after differentiation, myotubes derived from T2DM-patient myoblasts retain an altered myokine secretion distinct from that of non-diabetic myotubes (Ciaraldi et al., 2016). Although the mechanisms underlying the deteriorated function of myoblasts in DM have not been fully elucidated, several factors have been reported to inhibit myogenic differentiation. Co-culture with adipocytes increases interleukin (IL)-6 expression in myoblasts and attenuates their differentiation into myotubes (Seo et al., 2019). High ambient glucose suppresses the myogenesis of myoblasts by increasing the repressive myokine, myostatin, and decreasing myogenic transcription factors, MyoD and myogenin (Grzelkowska-Kowalczyk et al., 2013). Palmitic acid, a saturated fatty acid, blocks myotube formation by downregulating MyoD and myogenin (Saini et al., 2017). These findings demonstrate that diabetic factors including adipokines, glucose, and fatty acids are inhibitory factors for myoblast differentiation.

We recently identified myogenetic oligodeoxynucleotides (myoDNs), which are 18-base single-strand nucleotides that promote myoblast differentiation (Nihashi et al., 2020; Shinji et al., 2021). One of the myoDNs, iSN04, is directly incorporated into myoblasts and serves as an aptamer that physically interacts with nucleolin (Shinji et al., 2021). Nucleolin has been known to target the untranslated region of p53 mRNA to interfere with its translation (Takagi et al., 2005; Chen et al., 2012). In myoblasts, iSN04 antagonizes nucleolin, rescues p53 protein levels, and eventually facilitates myotube formation (Shinji et al., 2021). In this study, we aimed to determine that iSN04 recovers the deteriorated differentiation of myoblasts isolated from patients with DM. This study presents iSN04 as a potential nucleic acid drug targeting myoblasts for the prevention and therapy of muscle wasting in patients with DM.

## Materials and Methods

### Chemicals

All phosphodiester bonds of iSN04 (5′-AGA TTA GGG TGA GGG TGA-3′) were phosphorothioated to increase resistance to nucleases. Phosphorothioated iSN04 was synthesized and HPLC-purified (GeneDesign, Osaka, Japan), then was dissolved in endotoxin-free water as previously reported (Shinji et al., 2021). Palmitic acid (Wako, Osaka, Japan), which is the most abundant (occupying 20–30%) saturated fatty acid in human (Gesteiro et al., 2019), was dissolved in chloroform to prepare a high concentration stock (600 mM) to decrease treatment volume to myoblasts (Aguer et al., 2010). An equal volume of endotoxin-free water or chloroform, without the test chemicals, served as negative controls.

### Cell Culture

We purchased and used commercially available human myoblast (hMB) stocks (Lonza, Walkersville, MD, United States) isolated from healthy subjects (CC-2580) including a 26-year-old male (H26M; lot 18TL211617, August 2018), a 35-year-old female (H35F; lot 0000483427, June 2015), and a 35-year-old male (H35M; lot 0000650386, August 2017), from patients with T1DM (CC-2900) including an 81-year-old male (I81M; lot 0000211092, November 2010) and an 89-year-old female (I89F; lot 0000191810, August 2010), and from patients with T2DM (CC-2901) including a 68-year-old male (II68M; lot 0000211384, November 2010) and an 85-year-old female (II85F; lot 0000219206, January 2011). Detailed information of each hMB is described in certificate of analysis^1^. The hMBs were maintained in Skeletal Muscle Growth Media-2 (CC-3245; Lonza) as a growth medium for hMBs (hMB-GM). The murine myoblast cell line C2C12 (DS Pharma Biomedical, Osaka, Japan) was maintained in a growth medium for C2C12 cells (C2-GM) consisting of DMEM (Nacalai, Osaka, Japan) with 10% fetal bovine serum and a mixture of 100 units/ml penicillin and 100 μg/ml streptomycin (PS) (Nacalai). hMBs and C2C12 cells were differentiated in a differentiation induction medium (DIM) consisting of DMEM with 2% horse serum (HyClone; GE Healthcare, Salt Lake City, UT, United States) and PS (Nihashi et al., 2019b; Shinji et al., 2021).

hMB-GM, C2-GM, and DIM with 5.6 mM D-glucose and 19.4 mM mannitol (hMB-GM-NG, C2-GM-NG, and DIM-NG) were used for normal-glucose culture, and those with 25 mM D-glucose (hMB-GM-HG, C2-GM-HG, and DIM-HG) were used for high-glucose culture as previously described (La Sala et al., 2015). In the experiments using high-glucose culture, hMBs were maintained in hMB-GM-HG for a total of 6 days with passage every 3 days. The cells were then seeded on fresh dishes and differentiated in DIM-HG for 2 days. C2C12 cells were maintained in C2-GM-HG for a total of 4 days with passage every 2 days. The cells were then seeded on fresh dishes and differentiated in DIM-HG for 4 days. In the palmitic acid experiments, hMBs were maintained in hMB-GM-NG; then, the cells were seeded on fresh dishes and differentiated in DIM-NG with palmitic acid at an optimal concentration of 200 μM (for H26M) or 600 μM (for H35M) for 2 days, according to a previous study (Aguer et al., 2010).

All cells were cultured in dishes or plates coated with collagen type I-C (Cellmatrix; Nitta Gelatin, Osaka, Japan) at 37°C with 5% CO_2_ throughout the experiments.

### Immunocytochemistry

hMBs in hMB-GM (1.5–2.5 × 10^5^ cells/dish optimized for 70% confluency in each cell stock) or C2C12 cells in C2-GM (10 × 10^5^ cells/dish) were seeded on 30-mm dishes. The following day, the medium was replaced with DIM containing iSN04 at an optimal concentration of 1 μM (for H26M in hMB-DIM), 3 μM (C2C12 cells), 10 μM (for H26M in GM, H35M, and II85M), or 30 μM (for H35F, I81M, I89F, and II68M). Optimal iSN04 concentration for each MB was defined as indicating the highest ratio of MHC^+^ cells without affecting cell number because the sensitivities to iSN04 differ among MBs. Immunocytochemistry of myoblasts was performed as previously described (Takaya et al., 2017; Nihashi et al., 2019a; Shinji et al., 2021). The myoblasts were fixed with 2% paraformaldehyde, permeabilized with 0.2% Triton X-100, and immunostained with 0.5 μg/ml mouse monoclonal anti-MHC antibody (MF20; R&D Systems, Minneapolis, MN, United States) and 1.0 μg/ml rabbit polyclonal anti-nucleolin antibody (ab22758; Abcam, Cambridge, United Kingdom). 0.1 μg/ml each of Alexa Fluor 488-conjugated donkey polyclonal anti-mouse IgG antibody and Alexa Fluor 594-conjugated donkey polyclonal anti-rabbit IgG antibody (Jackson ImmunoResearch, West Grove, PA, United States) were used as secondary antibodies. Cell nuclei were stained with DAPI (Nacalai). Fluorescent images were captured using EVOS FL Auto microscope (AMAFD1000; Thermo Fisher Scientific, Waltham, MA, United States). The ratio of MHC^+^ cells was defined as the number of nuclei in the MHC^+^ cells divided by the total number of nuclei, and the fusion index was defined as the number of nuclei in the multinuclear MHC^+^ myotubes divided by the total number of nuclei; these were determined using ImageJ software (National Institutes of Health, United States).

### Quantitative Real-Time RT-PCR (qPCR)

Total RNA of the myoblasts was isolated using NucleoSpin RNA Plus (Macherey-Nagel, Düren, Germany) and reverse transcribed using ReverTra Ace qPCR RT Master Mix (TOYOBO, Osaka, Japan). qPCR was performed using GoTaq qPCR Master Mix (Promega, Madison, WI, United States) with StepOne Real-Time PCR System (Thermo Fisher Scientific). The amount of each transcript was normalized to that of human glyceraldehyde 3-phosphate dehydrogenase gene (*GAPDH*) and murine 18S ribosomal RNA (*Rn18s*). Results are presented as fold-change. The primer sequences are described in Supplementary Tables 1, 2.

### Statistical Analyses

Results are presented as the mean ± standard error. Statistical comparisons were performed using unpaired two-tailed Student’s *t*-test or multiple comparison test with Tukey–Kramer test following one-way analysis of variance. Statistical significance was set at *p* < 0.05.

## Results

### DM Deteriorates Myoblast Differentiation

**FIGURE 1:**
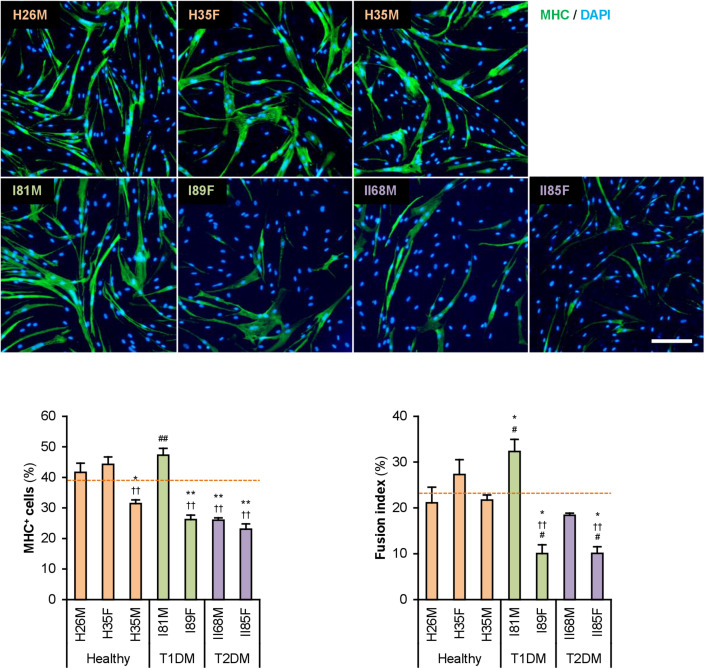
Attenuated myogenic differentiation of DM myoblasts. Representative immunofluorescent images of the hMBs differentiated in DIM-NG for 2 days. Scale bar, 200 μm. Ratio of MHC^+^ cells and multinuclear myotubes were quantified. Orange dashed lines indicate the mean values of H26M, H35F, and H35M. *p < 0.05, **p < 0.01 vs. H26M; ^††^*p* < 0.01 vs. H35F; ^#^*p* < 0.05, ^##^*p* < 0.01 vs. H35M (Tukey–Kramer test); *n* = 6.

The hMBs isolated from healthy subjects (H26M, H35F, and H35M), patients with T1DM (I81M and I89F), and patients with T2DM (II68M and II85F) were cultured in the hMB-GM-NG (Supplementary Figure 1). These hMBs varied in cell size and morphology, but DM-dependent hallmarks were not observed. The hMBs induced myogenic differentiation in DIM-NG, followed by immunostaining for MHC, a terminal differentiation marker of muscle cells. The ratio of MHC^+^ cells and multinuclear myotubes was quantified on days 0, 2, and 4 of differentiation (Supplementary Figure 2). On day 2 (Figure 1), the ratio of MHC^+^ cells of H35M was lower than that of H26M and H35F, indicating the individuality of myogenesis among healthy subjects. I81M differentiated to the same extent as H26M and H35F, but I89F, II68M, and II85F exhibited deteriorated differentiation. In particular, I89F and II85F were exacerbated in myotube formation compared to all healthy subjects. These results indicate that myoblast differentiation is aggravated in patients with DM.

**FIGURE 2:**
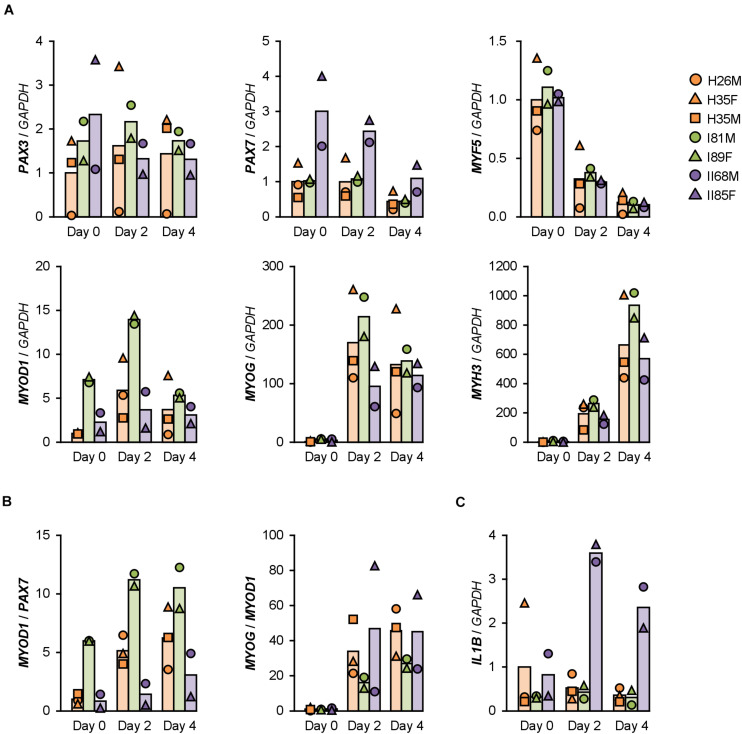
Gene expression patterns altered in DM myoblasts. **(A–C)** qPCR results of gene expression in the hMBs differentiated in DIM-NG on days 0, 2, and 4. Bars indicate mean values of each group. The mean value of healthy myoblasts on day 0 was set to 1.0 for each gene.

Gene expression patterns in hMBs were examined using qPCR (Figure 2A). Among undifferentiated myoblast markers, *PAX7* was expressed 2–3 times higher in T2DM myoblasts throughout differentiation, but *PAX3* and *MYF5* were not. A myogenic transcription factor, *MYOD1*, was highly induced in T1DM myoblasts, but a terminal transcription factor, myogenin (*MYOG*), was not. The mRNA levels of embryonic MHC (*MYH3*) were not significantly different among hMBs. The transcription levels of these genes frequently vary among patients, which reflects individual differences. During myogenic differentiation, the ratios of Pax7, MyoD, and myogenin are critically important. Proliferating myoblasts express both Pax7 and MyoD, but not myogenin. At the initial stage of differentiation, Pax7 disappears, and MyoD drives myogenin transcription. In terminally differentiated myocytes, MyoD decreases, and myogenin becomes a dominant transcription factor (Dumont et al., 2015). qPCR data indicated that *MYOD1*/*PAX7* and *MYOG*/*MYOD1* ratios were lower in T2DM and T1DM myoblasts than those in healthy myoblasts (Figure 2B), demonstrating a delayed shift of myogenic transcription factors in DM patients. This may be one of the reasons for the deteriorated differentiation of DM myoblasts.

### ILs Are Induced in T2DM Myoblasts

The mRNA levels of atrogin-1 (*FBXO32*), MuRF-1 (*TRIM63*), myostatin (*MSTN*), and myostatin receptor (*ACVR2B*), which are involved in ubiquitin-proteasome-mediated muscle atrophy (Bodine et al., 2001; Lokireddy et al., 2011), were not different among the hMBs. In contrast, transcription of the myostatin antagonist, follistatin (*FST*), was flat in T1DM myoblasts during differentiation (Supplementary Figure 3).

Sterol regulatory element-binding proteins (*SREBF1* and *SREBF2*), fatty acid synthase (*FASN*), insulin receptor substrates (*IRS1* and *IRS2*), glucose transporter 4 (*SLC2A4*), mitochondrial carnitine palmitoyltransferase 2 (*CPT2*), and thioredoxin interacting protein (*TXNIP*) are insulin resistance-related factors and involved in differentiation and fatty acid metabolism of muscle cells (Parikh et al., 2007; Kato et al., 2008; Lecomte et al., 2010; Boufroura et al., 2018). However, their mRNA levels were not significantly altered in T2DM myoblasts (Supplementary Figure 4).

Type 2 DM myoblasts have been reported to display abnormal inflammatory responses (Green et al., 2011). Indeed, mRNA levels of *IL1B* were 6–7 times higher in T2DM myoblasts than those in healthy myoblasts on days 2 and 4 (Figure 2C). In contrast, inflammatory factors, NF-κB p50 (*NFKB1*) and p65 (*RELA*) subunits, TNF-α (*TNF*), interferon γ (*IFNG*), and *IL6* were not upregulated in T2DM myoblasts. Although *IL8* (*CXCL8*) levels were high in H26M on day 0, T2DM myoblasts exhibited higher *IL8* mRNA levels than those did healthy myoblasts (Supplementary Figure 5). It has been reported that IL-1β inhibits insulin-like growth factor (IGF)-dependent myoblast differentiation (Broussard et al., 2004), and interleukin-8 (IL-8) is secreted from insulin-resistant myotubes (Bouzakri et al., 2011). Thus, the upregulation of IL-1β and IL-8 potentially impaired the shift in myogenic transcription factors and subsequent differentiation of T2DM myoblasts.

### myoDN Recovers Differentiation of DM Myoblasts

**FIGURE 3:**
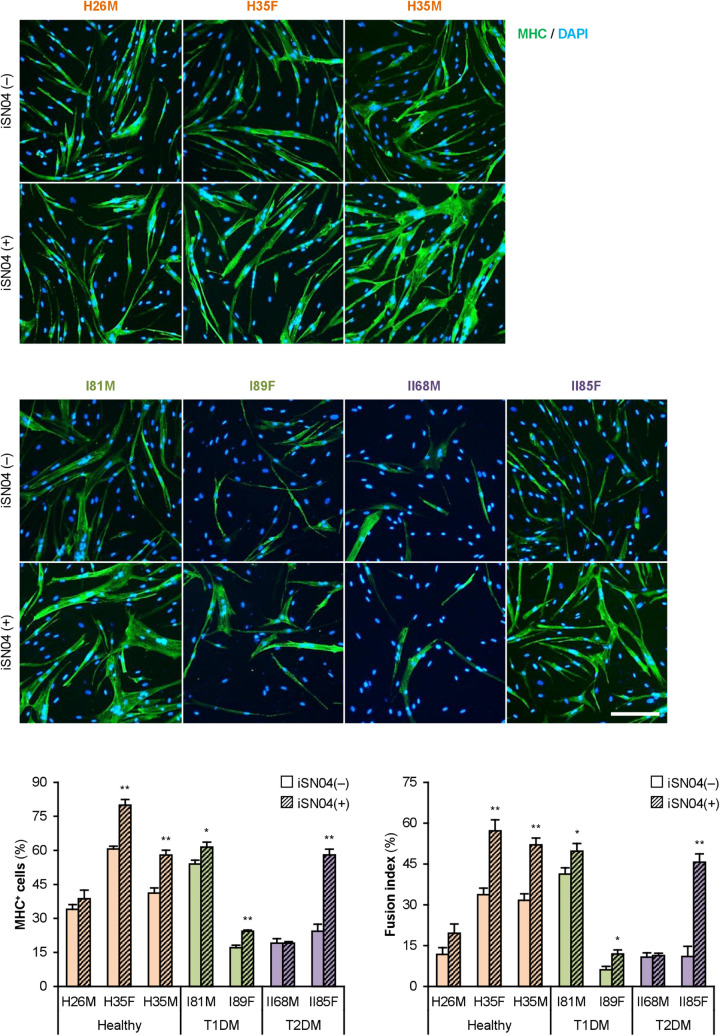
iSN04 recovers the differentiation of DM myoblasts. Representative immunofluorescent images of the hMBs differentiated in DIM-NG with iSN04 for 2 days. Scale bar, 200 μm. Ratio of MHC^+^ cells and multinuclear myotubes were quantified. *p < 0.05, **p < 0.01 vs. iSN04(–) in each group (Student’s *t*-test); *n* = 4–6.

**FIGURE 4:**
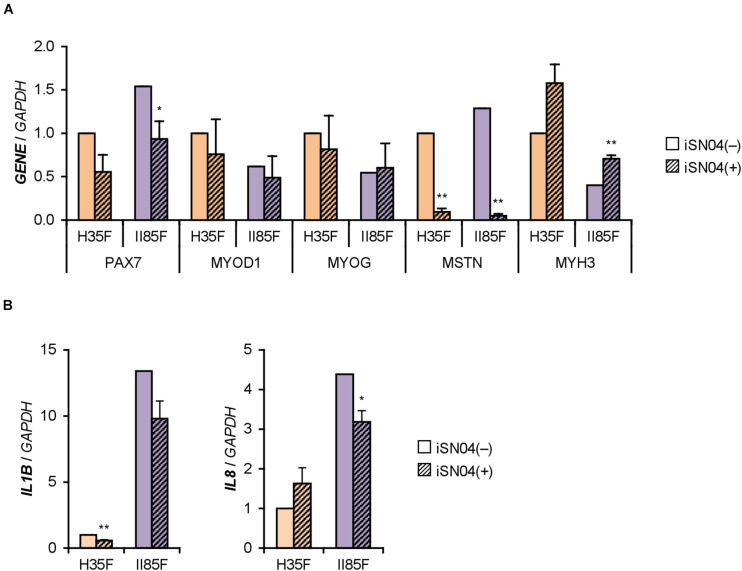
iSN04 suppresses myostatin expression. **(A,B)** qPCR results of gene expression in the H35F and II85F myoblasts differentiated in DIM-NG with iSN04 for 2 days. Mean value of H35F-iSN04(–) group was set to 1.0 for each gene. *p < 0.05, **p < 0.01 vs. iSN04(–) in each myoblast (Student’s *t*-test); *n* = 3.

We recently identified the single-strand myogenetic oligodeoxynucleotides (myoDNs) that promote myoblast differentiation by antagonizing nucleolin (Shinji et al., 2021). To assess the applicability of myoDN to DM myoblasts, the hMBs used in this study were treated with iSN04, which exhibits the highest myogenetic activity among the myoDNs. iSN04 significantly facilitated the differentiation and myotube formation of H35F, H35M, I81M, I89F, and II85F (Figure 3). In particular, iSN04 recovered the attenuated differentiation of II85F to almost the same extent as that of healthy myoblasts. iSN04 did not affect the differentiation of H26M in DIM, but significantly promoted myotube formation in hMB-GM (Supplementary Figure 6). In contrast, differentiation of II68M was not altered by iSN04, suggesting the distinct sensitivity or efficacy of iSN04 among individuals. These results indicate that iSN04 is able to recover the deteriorated differentiation of DM myoblasts. qPCR revealed that iSN04 treatment significantly reduced *PAX7* and *MSTN* mRNA levels in II85F, resulting in the recovery transcription of *MYH3* (Figure 4A). An iSN04-dependent decrease in *MSTN* expression was also detected in H35F. Furthermore, iSN04 significantly suppressed the *IL1B* levels in H35F and the *IL8* levels induced in II85F (Figure 4B). These results indicate that iSN04 facilitates the differentiation in both healthy and diabetic myoblasts, in part, by modulating the expression of cytokines including myostatin and ILs.

### myoDN Recovers the Myoblast Differentiation Impaired by Excessive Glucose

**FIGURE 5:**
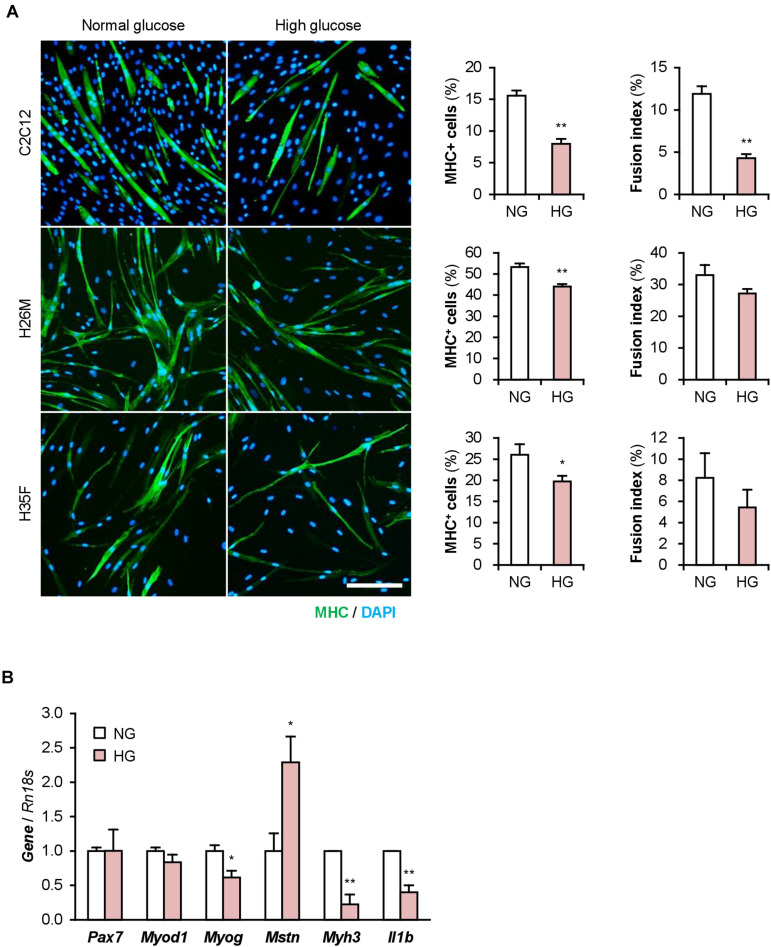
High glucose concentration deteriorates myoblast differentiation. **(A)** Representative immunofluorescent images of the C2C12, H26M, and H35F myoblasts differentiated in DIM-NG or -HG. Scale bar, 200 μm. Ratio of MHC^+^ cells and multinuclear myotubes were quantified. *p < 0.05, **p < 0.01 vs. NG (Student’s *t*-test); *n* = 4–6. **(B)** qPCR results of myogenic gene expression in the C2C12 cells differentiated in DIM for 1 day. Mean value of NG group was set to 1.0 for each gene. *p < 0.05, **p < 0.01 vs. NG (Student’s *t*-test); *n* = 3.

The DM myoblasts used in this study were isolated from elderly patients (68, 81, 85, and 89-year-old) whose ages were significantly higher than those of the healthy subjects (26, 35, and 35-year-old) (*p* < 0.01; Student’s *t*-test). Aging is a factor that compromises myoblast function (Fukada, 2018; McCormick and Vasilaki, 2018). Aged myoblasts are impaired to differentiate into myogenic lineage and induced to fibrogenic lineage due to the activated canonical Wnt signaling pathway (Brack et al., 2007). Therefore, aging possibly affected the deteriorated differentiation of the DM myoblasts used in this study. To investigate the impact of DM without aging on myogenesis, we cultured and differentiated C2C12 myoblast cell line in a high glucose concentration mimicking hyperglycemia. C2C12 cells maintained in high-glucose media exhibited a decreased ratio of MHC^+^ cells and myotubes (Figure 5A). qPCR revealed that high-glucose culture significantly induced *Mstn* and suppressed *Myog* and *Myh3* expression in C2C12 cells on differentiation day 1 (Figure 5B). It is noteworthy that *Il1b* mRNA levels were not elevated by excessive glucose. High-glucose culture also significantly abrogated the myogenesis of H26M and H35F (Figure 5A). These data demonstrated that excessive glucose is one of the age-independent factors for the deterioration of myoblast differentiation.

**FIGURE 6:**
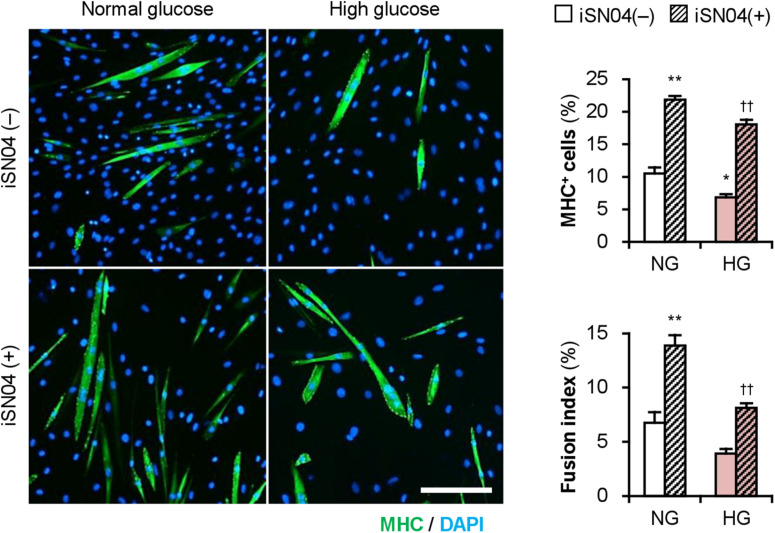
iSN04 recovers the myoblast differentiation impaired by excessive glucose. Representative immunofluorescent images of the C2C12 cells differentiated in DIM with iSN04 for 4 days. Scale bar, 200 μm. Ratio of MHC^+^ cells and multinuclear myotubes were quantified. *p < 0.05, **p < 0.01 vs. NG-iSN04(–); ^††^*p* < 0.01 vs. HG-iSN04(–) (Tukey–Kramer test); *n* = 4.

Importantly, iSN04 treatment significantly recovered myogenic differentiation and myotube formation in C2C12 cells exposed to high glucose concentrations (Figure 6). This result corresponds well with the phenotype of the iSN04-treated T2DM myoblasts, indicating that myoDNs are potential candidates for nucleic acid drugs that activate myoblasts in hyperglycemic patients.

### myoDN Recovers the Myoblast Differentiation Impaired by Palmitic Acid

**FIGURE 7:**
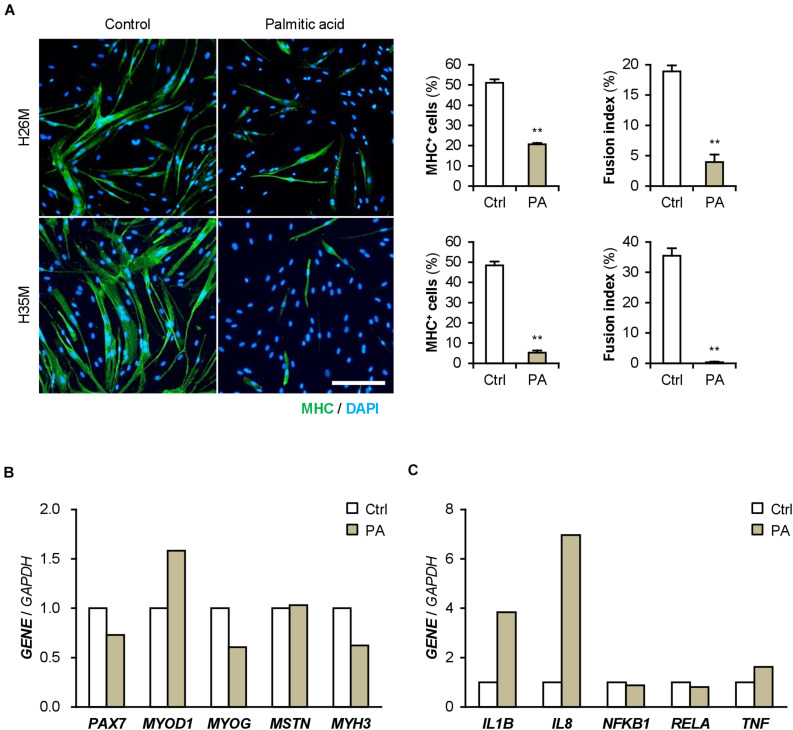
Palmitic acid (PA) deteriorates myoblast differentiation. **(A)** Representative immunofluorescent images of the H26M and H35F myoblasts differentiated in DIM-NG with PA for 2 days. Scale bar, 200 μm. Ratio of MHC^+^ cells and multinuclear myotubes were quantified. **p < 0.01 vs. control (Student’s *t*-test); *n* = 4–6. **(B,C)** qPCR results of gene expression in the H35M myoblasts differentiated in DIM-NG with palmitic acid for 2 days. Mean value of control group was set to 1.0 for each gene; *n* = 1.

Patients with T2DM are frequently present with hyperlipidemia. Palmitic acid is the most abundant intravital fatty acid, which is involved in insulin resistance and C2C12 cell differentiation (Yang et al., 2013; Saini et al., 2017). To examine the impact of excessive fatty acids on hMBs, H26M and H35M were induced to differentiate in DIM-NG with palmitic acid. In both hMBs, palmitic acid significantly impaired myogenic differentiation and myotube formation (Figure 7A). qPCR showed that palmitic acid decreased the *MYOG*/*MYOD1* ratio, resulting in lower *MYH3* expression in H35M (Figure 7B). Palmitic acid also upregulated *IL1B* and *IL8* mRNA levels without altering *NFKB1*, *RELA*, and *TNF* (Figure 7C), which recapitulated the phenotype of T2DM myoblasts. These results indicate that excessive fatty acids is another age-independent factor to inhibit myoblast differentiation by inducing inflammatory cytokines.

**FIGURE 8:**
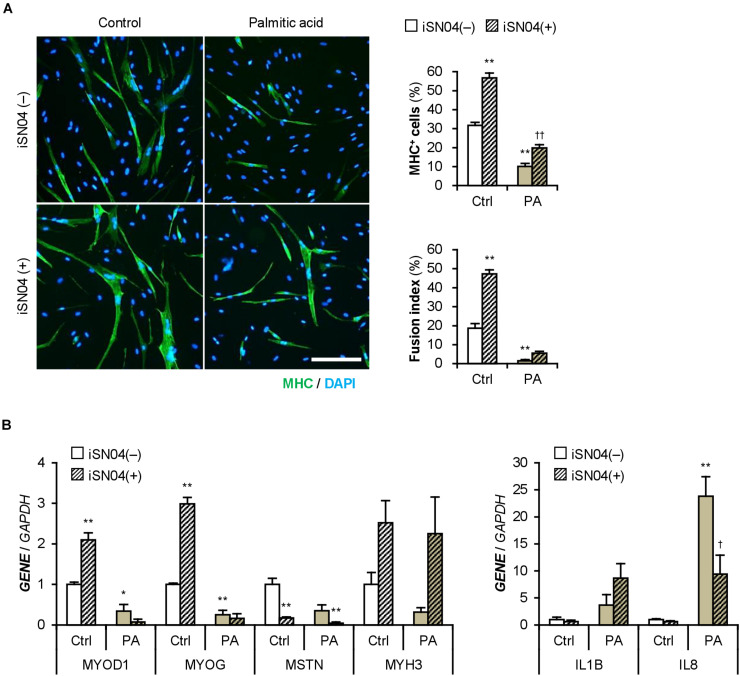
iSN04 recovers the myoblast differentiation impaired by excessive palmitic acid. **(A)** Representative immunofluorescent images of the H35M myoblasts differentiated in DIM-NG with palmitic acid and iSN04 for 2 days. Scale bar, 200 μm. Ratio of MHC^+^ cells and multinuclear myotubes were quantified. **p < 0.01 vs. control-iSN04(–); ^††^*p* < 0.01 vs. PA-iSN04(–) (Tukey–Kramer test); *n* = 6. **(B)** qPCR results of gene expression in the H35M myoblasts differentiated as in panel **(A)**. Mean value of control-iSN04(–) group was set to 1.0 for each gene. *p < 0.05, **p < 0.01 vs. control-iSN04(–); ^†^*p* < 0.05 vs. palmitic acid-iSN04(–) (Tukey–Kramer test); *n* = 3.

iSN04 treatment significantly improved the differentiation into MHC^+^ cells from palmitic acid-treated H35M (Figure 8A). As shown in Figure 8B, iSN04 induced *MYOD1* and *MYOG* expression under basal conditions, but not in the presence of palmitic acid. In contrast, iSN04 significantly reduced *MSTN* mRNA levels regardless of the presence of palmitic acid. iSN04 further suppressed palmitic acid-induced *IL8* transcription. These results show that myoDNs conceivably recover myoblast differentiation attenuated by excessive fatty acids in hyperlipidemic patients.

## Discussion

This study provides evidence that the myoDN, iSN04, ameliorates the differentiation of DM myoblasts, and presents a novel therapeutic strategy for muscle wasting in patients with DM. Dysfunction of DM myoblasts is caused by various pathophysiological factors such as inflammation and transcriptional disorders (D’Souza et al., 2013; Teng and Huang, 2019), and impaired myogenesis can be one of the reasons for muscle atrophy. A decreased number of satellite cells has been reported in patients with T1DM (D’Souza et al., 2016). Our results further showed the impaired myogenic ability of T1DM myoblasts with a delayed shift to myogenin-dominant transcription. A similar attenuation of myogenesis has been reported in T2DM myoblasts (Henriksen et al., 2017, 2019). The T2DM myoblasts used in this study exhibited a diminished ratio of MyoD/Pax7 and elevated levels of IL-1β and IL-8, which may contribute to the incompetent differentiation. As many patients with T2DM are accompanied by hyperlipidemia in addition to hyperglycemia, surplus glucose and fatty acids are considered the major molecules that interfere with myoblast differentiation. In this study, excessive glucose upregulated myostatin and downregulated myogenin and MHC in C2C12 cells, which is consistent with that reported in previous studies (Grzelkowska-Kowalczyk et al., 2013; Jeong et al., 2013). Similarly, high-glucose culture inhibited myogenesis of plural healthy hMBs. This demonstrates that glucose is an independent factor for myoblast dysfunction, which modulates myogenic gene expression. However, high-glucose culture did not induce IL-1β. Palmitic acid inhibits myokine expression and C2C12 cell differentiation (Yang et al., 2013; Saini et al., 2017). We showed that palmitic acid abrogates the differentiation of healthy hMBs by upregulating IL-1β and IL-8. IL-1β is known to inhibit IGF-induced myogenin expression and myogenesis (Broussard et al., 2004). IL-8 is a chemokine that contributes to insulin resistance in patients with T2DM (Kim et al., 2006; Samaras et al., 2010) and is also a myokine released from skeletal muscle cells. Insulin-resistant human myotubes secrete higher levels of IL-8 (Bouzakri et al., 2011). The role of IL-8 in myoblast differentiation remains controversial. IL-8-neutralizing antibody impairs the differentiation of hMBs (Polesskaya et al., 2016). In contrast, IL-8 treatment decreases the myogenin/MyoD ratio and embryonic MHC expression in rat myoblasts (Milewska et al., 2019). An appropriate level of IL-8 is important for normal myogenesis. Perturbation of IL-8 in T2DM and palmitic acid-cultured myoblasts may be linked to deteriorated differentiation. The mechanism of IL induction in myoblasts remains unclear. NF-κB p65 and TNF-α have been reported to be elevated in T2DM myoblasts (Green et al., 2011). However, in this study, mRNA levels of these genes were not altered by T2DM or palmitic acid. The signaling pathway of fatty acid-dependent IL induction needs to be clarified in further studies.

In addition to inflammation, abnormal protein metabolism is another important factor for diabetic skeletal muscle wasting. In skeletal muscle tissue, T1DM decreases protein synthesis and T2DM enhances autophagy-mediated protein degradation (Sala and Zorzano, 2015). A recent study reported the dysregulated autophagy in T2DM myoblasts (Henriksen et al., 2019). However in this study, expression of proteasome-related genes such as atrogin-1 and MuRF-1 were not altered between healthy and DM myoblasts during short-term differentiation. The effects of T2DM on protein degradation might emerge in the fully maturated myofibers, suggesting the limitation of cultured myoblasts as a model of muscle wasting in patients with DM. Present results need to be carefully interpreted in clinical settings.

This study proved that iSN04 can recover the deteriorated myogenesis of DM myoblasts, in addition to facilitating the differentiation of healthy myoblasts. Although myoDNs, including iSN04, can be potential drug seeds for muscle wasting in patients with DM, the effect of iSN04 should be established using extensive patient-derived myoblasts for clinical application. For instance, the sensitivities to iSN04 were individually different among hMBs. iSN04 is incorporated into the cytoplasm and physically interacts with and interfere with a multifunctional phosphoprotein, nucleolin (Shinji et al., 2021). Nucleolin (*NCL*) mRNA levels were similar among the hMBs used in this study (Supplementary Figure 7A), and subcellular localization of nucleolin was not different between insensitive H26M and sensitive H35F throughout differentiation (Supplementary Figure 7B). Post-translational phosphorylation or glycosylation is indispensable for nucleolin function (Barel et al., 2001; Losfeld et al., 2009). This suggests that the modification of nucleolin may vary among individuals and may be related to iSN04 sensitivity. The precise role of nucleolin in myoblasts remains unclear. One study reported that a moderate knockdown of nucleolin by miR-34b promotes myoblast differentiation (Tang et al., 2017). We found that iSN04 serves as a nucleolin antagonist and increases p53 protein levels to promote myoblast differentiation (Shinji et al., 2021) because nucleolin binds to p53 mRNA to inhibit its translation (Takagi et al., 2005; Chen et al., 2012). However, inhibition of p53 translation is considered to be a part of the multifunction of nucleolin in myoblasts. In cancer cells, nucleolin competitively interacts with NF-κB essential modulator (NEMO), resulting in the downregulation of NF-κB activity. The established nucleolin aptamer AS1411 forms the NEMO-nucleolin-AS1411 complex to block the transcriptional activity of NF-κB (Girvan et al., 2006). We have already confirmed that AS1411 promotes myoblast differentiation as well as iSN04 (Shinji et al., 2021). Thus, iSN04 possibly inhibits NF-κB activity by associating the NEMO-nucleolin-iSN04 complex. NF-κB has been known to impair myogenesis by upregulating Pax7 and myostatin (Wang et al., 2007; He et al., 2013; Ono and Sakamoto, 2017). Inactivation of NF-κB by iSN04 can be assumed to downregulate myostatin and IL-8 in T2DM and palmitic acid-cultured myoblasts. Investigation of anti-inflammatory effects of iSN04 and AS1411 in myoblasts should be an important subject to reveal their action mechanism and to establish the myoDNs as nucleic acid drugs for skeletal muscle loss in patients with DM.

## Conclusion

The differentiation abilities of myoblasts deteriorated with dysregulation of myogenic and inflammatory gene expression due to DM, glucose, or palmitic acid. A myoDN, iSN04, recovered impaired myogenesis by modulating gene expression, especially by decreasing myostatin and IL-8. iSN04 could be a potential drug candidate for muscle wasting in patients with DM by directly targeting myoblasts.

## Data Availability Statement

The raw data supporting the conclusions of this article will be made available by the authors, without undue reservation.

## Author Contributions

TT and SN designed the study. TT wrote the manuscript. SN performed the experiments and analyses. SY and TS provided the materials and supervised the study. All authors contributed to the article and approved the submitted version.

## Conflict of Interest

Shinshu University has been assigned the invention of iSN04 by TT, Koji Umezawa, and TS, and Japan Patent Application 2018-568609 has been filed on February 15, 2018. The remaining authors declare that the research was conducted in the absence of any commercial or financial relationships that could be construed as a potential conflict of interest.

## Footnotes

1 https://bioscience.lonza.com/lonza_bs/CH/en/coa/search
