# Supplementary material for: Myogenetic Oligodeoxynucleotide (myoDN) Recovers the Differentiation of Skeletal Muscle Myoblasts Deteriorated by Diabetes Mellitus
Source: Front Physiol. 2021 May 24;12:679152. doi: 10.3389/fphys.2021.679152 (PMC8181739; doi:10.3389/fphys.2021.679152)
Supplement: Supplementary file 1 [file Data_Sheet_1.PDF]

frontiers

Supplementary Material

Supplementary Table S1. qPCR primer sequences for human genes.

Gene	Sequence (5'-3')	Reference
ACVR2B	GGCTGCTGGCTAGATGACTT<br>AAGCGTTCGTTGCAGAAGTT	Senanayake et al., 2012
CPT2	AGCCTCTCTTGAATGATGGCC<br>GATAGGTACATATCAAACCAGGG	Boufroura et al., 2018
FASN	CTTCCGAGATTCCATCCTACGC<br>TGGCAGTCAGGCTCACAAACG	Sun et al., 2018
FBXO32	CCCAAGGAAAGAGCAGTATGGAGA<br>GGGTGAAAGTGAAACGGAGCA	D'Hulst et al., 2013
FST	TGCTCTGCCAGTTCATGG<br>CTTGACGGAGCCAGCAGT	Cheng et al., 2014
GAPDH	TGTCAAGCTCATTTCCTGGTA<br>GTGAGGGTCTCTCTCTTCCTCTTGT	Shinji et al., 2020
IFNG	AGGGAAGCGAAAAAGGAGTCA<br>GGACAACCATTACTGGGATGCT	Chege et al., 2010
IL1B	TCCCCAGCCCTTTTGTTGA<br>TTAGAACCAAATGTGGCCGTG	Sjolinder et al., 2012
IL6	CGGGAACGAAAGAGAAGCTCTA<br>GAGCAGCCCCAGGGAGAA	Grosse et al., 2012
IL8 (*CXCL8*)	TGGCAGCCTTCCTGATTTCT<br>GGGTGGAAAGGTTTGGAGTATG	Grosse et al., 2012
IRS1	TATGCCAGCATCAGTTTCCA<br>TTGCTGAGGTCATTTAGGTCTT	Zhao et al., 2017
IRS2	TTCTTGTCCCACCACTTGAA<br>CTGACATGTGACATCCTGGTG	Zhao et al., 2017
MSTN	CTACAACGGAAACAATCATTACCA<br>GTTTCAGAGATCGGATTCCAGTAT	Bathgate et al., 2018
MYF5	AGAACTACTATAGCCTGCCGG<br>ATCTGTGGCATATACATTTGATACATCA	Zibat et al., 2010

MYH3	GGACAGGAAGAATGTGCTGAGATT<br>GCCTCTTGTAGGACTTGACTTTCAC	Shinji et al., 2021
MYOD1	TGCTCCGACGGCATGATGGAC<br>TCGACACCGCCGCACTCT	Shinji et al., 2021
MYOG	AACCCAGGGGATCATCTGCTCAC<br>GTTGGGCATGGTTTCATCTGGGAAG	Shinji et al., 2021
NCL	ATTGGTAGCAACTCCTGGTAAG<br>CACTGTCATCATCCTCCTCTTC	Shinji et al., 2021
NFKB1	CACAAGGCAGCAAATAGACG<br>GAGTTAGCAGTGAGGCACCA	Zhao et al., 2018
PAX3	AGGAAGGAGGCAGAGGAAAG<br>CAGCTGTTCTGCTGTGAAGG	Sato et al., 2019
PAX7	GACCCCTGCCTAACCACATC<br>GTCTCCTGGTAGCGGCAAAG	Shinji et al., 2021
RELA	CGCTTCTTCACACACTGGATTC<br>ACTGCCGGGATGGCTTCT	Grosse et al., 2012
SLC2A4	CATCCTGATGACTGTGGCTC<br>TCTCATCTGGCCCTAAATACT	Armoni et al., 2005
SREBF1	TCCCAGCCCCTCAGATACCAC<br>CCCATTGAGCAGCCAGACCAC	Yang et al., 2019
SREBF2	CCCTCACCACCCCTATCCAGA<br>CTCTTGCCCCATCATTACAGG	Yang et al., 2019
TNF	CCTGCCCCAATCCCTTTATT<br>CCCTAAGCCCCCAATTCTCT	Sjolinder et al., 2012
TP53	AGGCCTTGGAACTCAAGGAT<br>CCCTTTTTGGACTTCAGGTG	Henriksen et al., 2017
TRIM63	AAACAGGAGTGCTCCAGTCGG<br>CGCCACCAGCATGGAGATACA	D'Hulst et al., 2013
TXNIP	GGCTAAAGTGCTTTGGATGC<br>AGGTCTCATGATCACCATCTCA	Houshmand-Oeregaard et al., 2017

Supplementary Table S2. qPCR primer sequences for murine genes.

Il1b	TTGACGGACCCCAAAAGATG<br>CAGGACAGCCCAGGTCAAA	Qu et al., 2009
Mstn	ATGGCCATGATCTTGCTGTA<br>CCTTGACTTCTAAAAAGGGATTCA	Han et al., 2010
Myh3	CACCTGGAGAGGATGAAGAAGAA<br>AGGACTTGACTTTCACTTGGAGTTTATC	This study
Myod1	GATGGCATGATGGATTACAGCGGC<br>GTGGAGATGCGCTCCACTATGCTG	This study
Myog	CCCTATTTCTACCAGGAGCCCCAC<br>GCGCAGGATCTCCACTTTAGGCAG	Watanabe et al., 2011
Pax7	CGCGTCCAGGTCTGGTTCAGTAAC<br>GTACTGTGCTGCCTCCATCTTGGG	This study
Rn18s	CGCACGGCCGGTACAGTGAAACTG<br>CACCCGTGGTCACCATGGTAGGCA	Nihashi et al., 2019

Supplementary Figure Legends

Supplementary Figure S1. The hMBs used in this study. Representative images of the hMBs maintained in hMB-GM-NG. Scale bars, 500 µm (×40) and 50 µm (×200).

Supplementary Figure S2. Attenuated myogenic differentiation of DM myoblasts. Ratio of MHC^+ cells and multinuclear myotubes of the hMBs differentiated in DIM-NG on days 0, 2, and 4. Bars indicate mean values of each group.

Supplementary Figure S3. qPCR results of muscle atrophic gene expression in the hMBs differentiated in DIM-NG on days 0, 2, and 4. Bars indicate mean values of each group. The mean value of healthy myoblasts on day 0 was set to 1.0 for each gene.

Supplementary Figure S4. qPCR results of metabolic gene expression in the hMBs differentiated in DIM-NG on days 0, 2, and 4. Bars indicate mean values of each group. The mean value of healthy myoblasts on day 0 was set to 1.0 for each gene.

Supplementary Figure S5. qPCR results of inflammatory gene expression in the hMBs differentiated in DIM-NG on days 0, 2, and 4. Bars indicate mean

values of each group. The mean value of healthy myoblasts on day 0 was set to 1.0 for each gene.

Supplementary Figure S6. iSN04 promotes the differentiation of H26M myoblasts in GM. Representative immunofluorescent images of the H26M differentiated in hMB-GM-NG with 10 µM iSN04 for two days. Scale bar, 200 µm. Ratio of MHC^+ cells and multinuclear myotubes were quantified. ** $p < 0.01$ vs. control (Student's *t*-test). $n = 6$.

Supplementary Figure S7. Expression and localization of nucleolin in the hMBs used in this study. **(A)** qPCR results of *NCL* expression in the hMBs differentiated in DIM-NG on days 0, 2, and 4. Bars indicate mean values of each group. The mean value of healthy myoblasts on day 0 was set to 1.0 for each gene. **(B)** Representative immunofluorescent images of the hMBs differentiated in hMB-DIM-NG. Scale bar, 50 µm.

Supplementary References

Armoni, M., Harel, C., Bar-Yoseph, F., Milo, S., Karnieli, E. (2005). Free fatty acids repress the GLUT4 gene expression in cardiac muscle via novel response elements. *J. Biol. Chem.* 280, 34786-34795. doi: 10.1074/jbc.M502740200

Bathgate, K. E., Bagley, J. R., Jo, E., Talmadge, R. J., Tobias, I. S., Brown, L. E., et al. (2018). Muscle health and performance in monozygotic twins with 30 years of discordant exercise habits. *Eur. J. Appl. Physiol.* 118, 2097-2110. doi: 10.1007/s00421-018-3943-7

Boufroura, F. Z., Le Bachelier, C., Tomkiewicz-Raulet, C., Schlemmer, D., Benoist, J. F., Grondin, P., et al. (2018). A new AMPK activator, GSK773, corrects fatty acid oxidation and differentiation defect in CPT2-deficient myotubes. *Hum. Mol. Genet.* 27, 3417-3433. doi: 10.1093/hmg/ddy254

Chege, D., Chai, Y., Huibner, S., McKinnon, L., Wachihi, C., Kimani, M., et al. (2010). *PLoS One* 5, e13077. doi: 10.1371/journal.pone.0013077

Cheng, J. C., Chang, H. M., Qiu, X., Fang, L., Leung, P. C. (2014). FOXL2-induced follistatin attenuates activin A-stimulated cell proliferation in human granulosa cell tumors. *Biochem. Biophys. Res. Commun.* 443, 537-542. doi: 10.1016/j.bbrc.2013.12.010

D'Hulst, G., Jamart, C., Van Thienen, R., Hespel, P., Francaux, M., Deldicque, L. (2013). Effect of acute environmental hypoxia on protein metabolism in human skeletal muscle. *Acta Physiol.* 208, 251-264. doi: 10.1111/apha.12086

Grosse, J., Wehland, M., Pietsch, J., Schulz, H., Saar, K., Hubner, N., et al. (2012). Gravity-sensitive signaling drives 3-dimensional formation of multicellular thyroid cancer spheroids. *FASEB J.* 26, 5124-5140. doi: 10.1096/fj.12-215749

Han, D. S., Huang, H. P., Wang, T. G., Hung, M. Y., Ke, J. Y., Chang, K. T., et al. (2010). Transcription activation of myostatin by trichostatin A in differentiated C2C12 myocytes via ASK1-MKK3/4/6-JNK and p38 mitogen-activated protein kinase pathways. *J. Cell. Biochem.* 111, 564-573. doi: 10.1002/jcb.22740

Henriksen, T. I., Davidsen, P. K., Pedersen, M., Schultz, H. S., Hansen, N. S., Larsen, T. J., et al. (2017). Dysregulation of a novel miR-23b/27b-p53 axis impairs muscle stem cell differentiation of humans with type 2 diabetes. *Mol. Metab.* 6, 770-779. doi: 10.1016/j.molmet.2017.04.006

Houshmand-Oeregaard, A., Hjort, L., Kelstrup, L., Hansen, N. S., Broholm, C., Gillberg, L., et al. (2017). DNA methylation and gene expression of TXNIP in adult offspring of women with diabetes in pregnancy. *PLoS One* 12, e0187038. doi: 10.1371/journal.pone.0187038

Nihashi, Y., Umezawa, K., Shinji, S., Hamaguchi, Y., Kobayashi, H., Kono, T., et al. (2019). Distinct cell proliferation, myogenic differentiation, and gene expression in skeletal muscle myoblasts of layer and broiler chickens. *Sci. Rep.* 9, 16527. doi: 10.1038/s41598-019-52946-4

Qu, P., Du, H., Wilkes, D. S., Yan, C. (2009). Critical roles of lysosomal acid lipase in T cell development and function. *Am. J. Pathol.* 174, 944-956. doi: 10.2353/ajpath.2009.080562

Sato, T., Higashioka, K., Sakurai, H., Yamamoto, T., Goshima, N., Ueno, M., et al. (2019). Core transcription factors promote induction of PAX3-positive skeletal muscle stem cells. *Stem Cell Reports* 13, 352-365. doi: 10.1016/j.stemcr.2019.06.006

Senanayake, U., Das, S., Vesely, P., Alzoughbi, W., Frohlich, L. F., Chowdhury, P., et al. (2012). miR-192, miR-194, miR-215, miR-200c and miR-141 are downregulated and their common target ACVR2B is strongly expressed in renal childhood neoplasms. *Carcinogenesis* 33, 1014-1021. doi: 10.1093/carcin/bgs126

Shinji, S., Nakamura, S., Nihashi, Y., Umezawa, K., and Takaya, T. (2020). Berberine and palmatine inhibit the growth of human rhabdomyosarcoma cells. *Biosci. Biotechnol. Biochem.* 84, 63-75. doi: 10.1080/09168451.2019.1659714

Shinji, S., Umezawa, K., Nihashi, Y., Nakamura, S., Shimosato, T., and Takaya, T. (2021). Identification of the myogenetic oligodeoxynucleotides (myoDNs) that promote differentiation of skeletal muscle myoblasts by targeting nucleolin. *Front. Cell Dev. Biol.* 8, 616706. doi: 10.3389/fcell.2020.616706

Sjolinder, M., Altenbacher, G., Wang, X., Gao, Y., Hansson, C., and Sjolinder, H. (2012). The meningococcal adhesin NhhA provokes proinflammatory responses in macrophages via toll-like receptor 4-dependent and -independent pathways. *Infect. Immun.* 80, 4027-4033. doi: 10.1128/IAI.00456-12

Sun, L., Yao, Y., Pan, G., Zhan, S., Shi, W., Lu, T., et al. (2018). Small interfering RNA-mediated knockdown of fatty acid synthase attenuates the proliferation and metastasis of human gastric cancer cells via the mTOR/Gli1 signaling pathway. *Oncol. Lett.* 16, 594-602. doi: 10.3892/ol.2018.8648

Watanabe, S., Hirai, H., Asakura, Y., Tastad, C., Verma, M., Keller, C., et al. (2011). MyoD gene suppression by Oct4 is required for reprogramming in myoblasts to produce induced pluripotent stem cells. *Stem Cells* 29, 505-513. doi: 10.1002/stem.598

Yang, H., Li, J., Yang, C., Liu, H., and Cao, Y. (2019). Multi-walled carbon nanotubes promoted lipid accumulation in human aortic smooth muscle cells. *Toxicol. Appl. Pharmacol.* 374, 11-19. doi: 10.1016/j.taap.2019.04.022

Zhao, H., Liu, F., Jia, R., Chang, H., Li, H., Miao, M., et al. (2017). MiR-570 inhibits cell proliferation and glucose metabolism by targeting IRS1 and IRS2 in human chronic myelogenous leukemia. *Iran. J. Basic Med. Sci.* 20, 481-488. doi: 10.22038/IJBMS.2017.8671

Zhao, M., Joy, J., Zhou, W., De, S., Wood, W. H., 3rd, Becker, K. G., et al. (2018). Transcriptional outcomes and kinetic patterning of gene expression in response to NF-κB activation. *PLoS Biol.* 16, e2006347. doi: 10.1371/journal.pbio.2006347

Zibat, A., Missiaglia, E., Rosenberger, A., Pritchard-Jones, K., Shipley, J., Hahn, H., et al. (2010). Activation of the hedgehog pathway confers a poor prognosis in embryonal and fusion gene-negative alveolar rhabdomyosarcoma. *Oncogene* 29, 6323-6330. doi: 10.1038/onc.2010.368

Figure S1

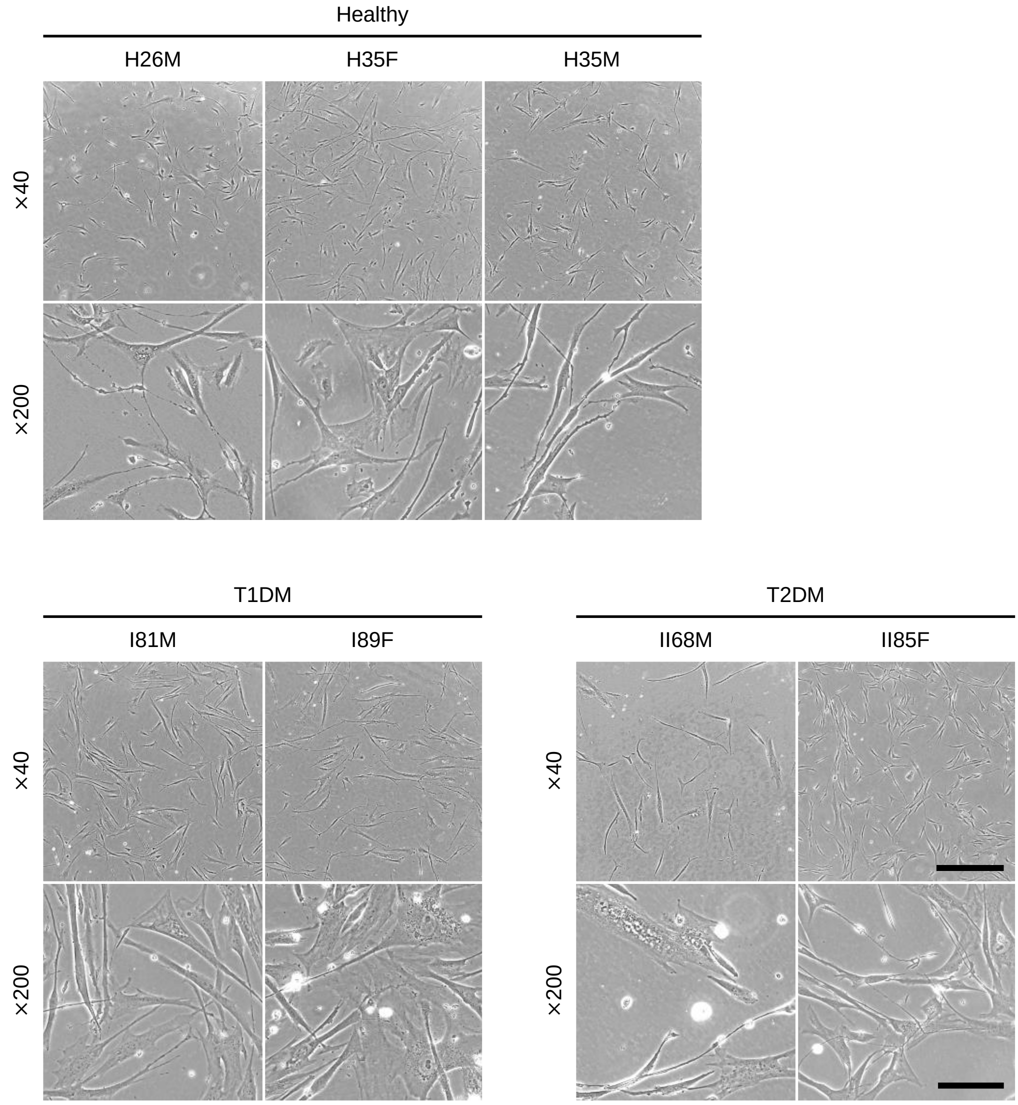
Healthy
H26M
H35F
H35M
×40
×200
T1DM
I81M
I89F
×40
×200
T2DM
II68M
II85F
×40
×200

Figure S2

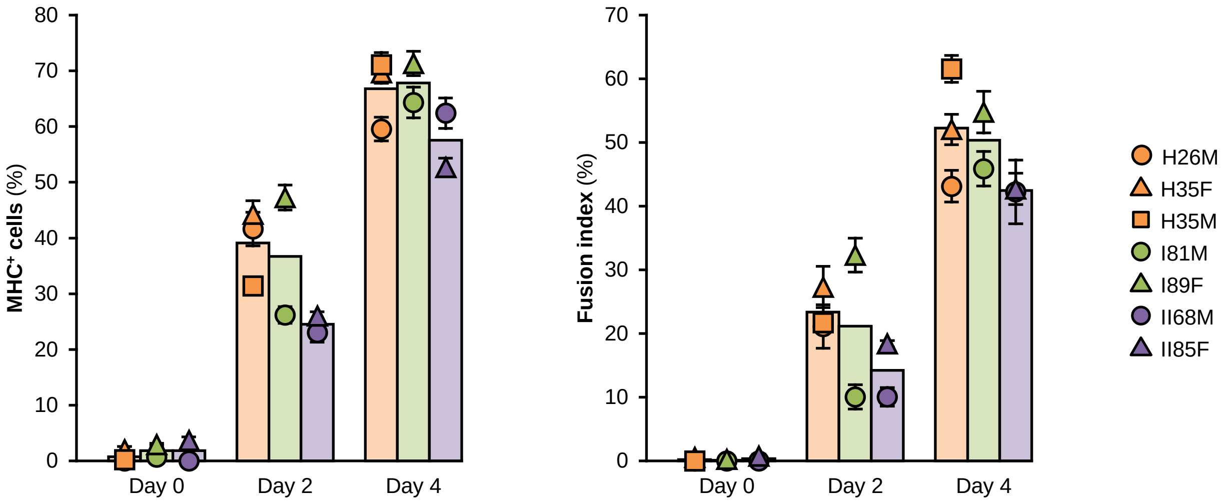
80
70
60
50
40
30
20
10
0
MHC⁺ cells (%)
Day 0
Day 2
Day 4
70
60
50
40
30
20
10
0
Fusion index (%)
Day 0
Day 2
Day 4
H26M
H35F
H35M
I81M
I89F
II68M
II85F

Figure S3

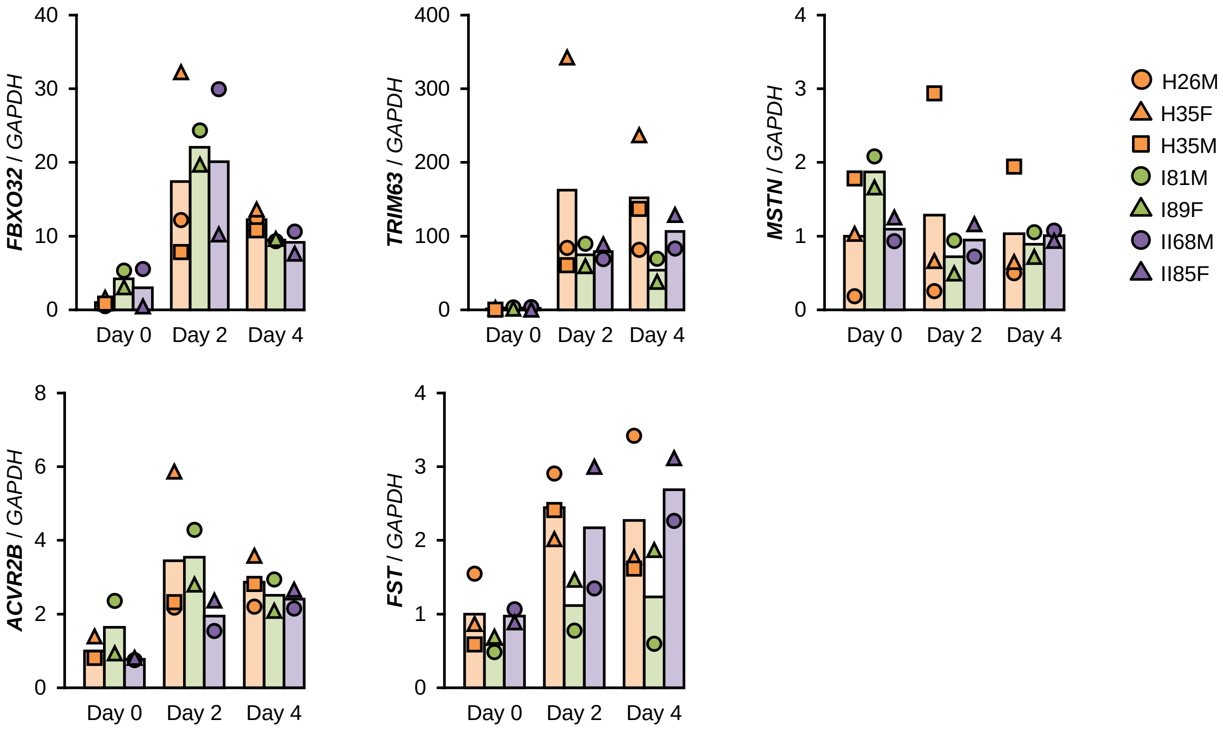
FBXO32 / GAPDH
TRIM63 / GAPDH
MSTN / GAPDH
ACVR2B / GAPDH
FST / GAPDH
Day 0
Day 2
Day 4
H26M
H35F
H35M
I81M
I89F
II68M
II85F

Figure S4

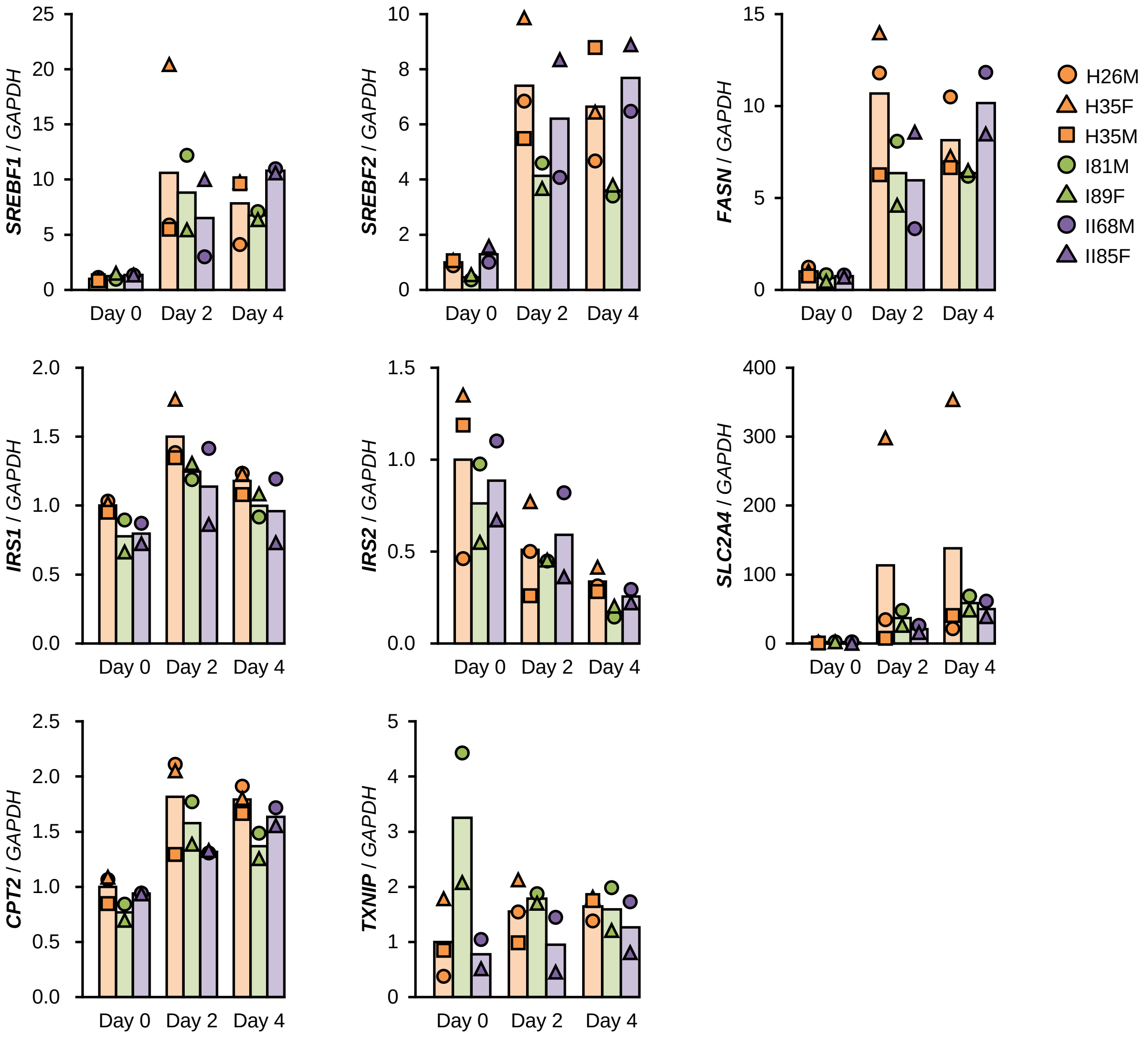

25
20
15
10
5
0
SREBF1 / GAPDH
Day 0
Day 2
Day 4
10
8
6
4
2
0
SREBF2 / GAPDH
Day 0
Day 2
Day 4
15
10
5
0
FASN / GAPDH
Day 0
Day 2
Day 4
H26M
H35F
H35M
I81M
I89F
II68M
II85F
2.0
1.5
1.0
0.5
0.0
IRS1 / GAPDH
Day 0
Day 2
Day 4
1.5
1.0
0.5
0.0
IRS2 / GAPDH
Day 0
Day 2
Day 4
400
300
200
100
0
SLC2A4 / GAPDH
Day 0
Day 2
Day 4
2.5
2.0
1.5
1.0
0.5
0.0
CPT2 / GAPDH
Day 0
Day 2
Day 4
5
4
3
2
1
0
TXNIP / GAPDH
Day 0
Day 2
Day 4

Figure S5

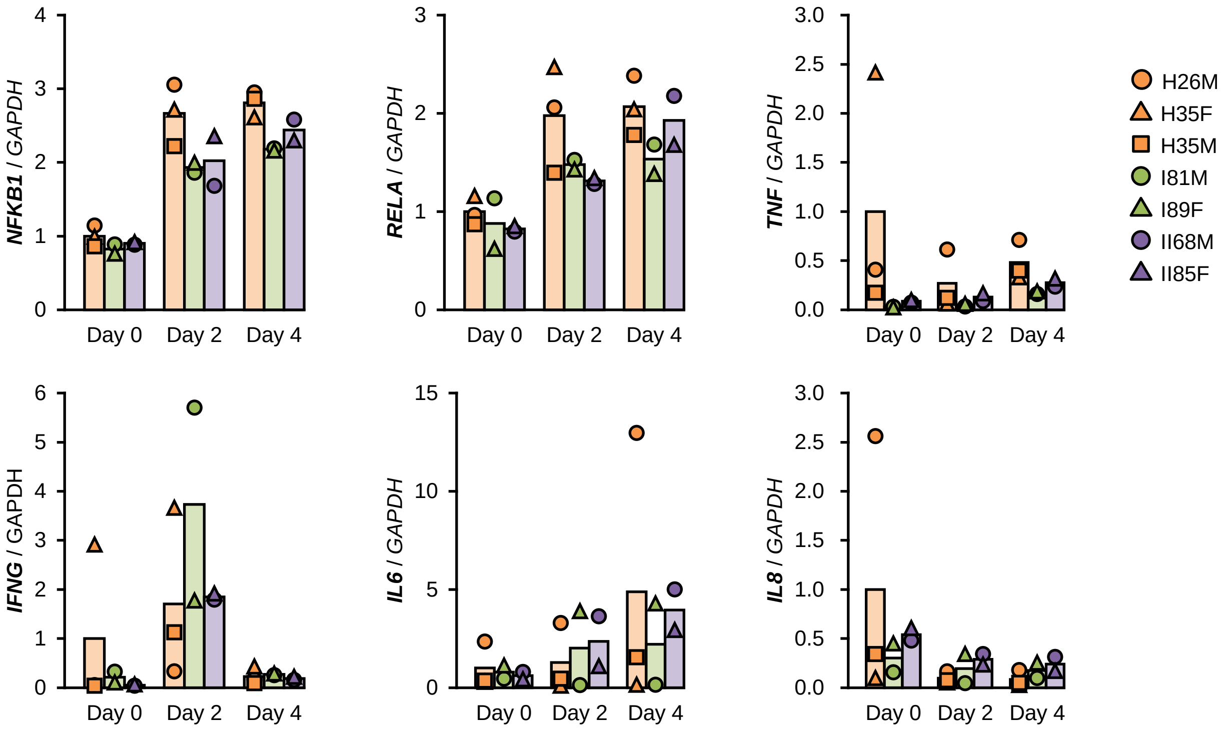

NFKB1 / GAPDH
RELA / GAPDH
TNF / GAPDH
IFNG / GAPDH
IL6 / GAPDH
IL8 / GAPDH
Day 0
Day 2
Day 4
H26M
H35F
H35M
I81M
I89F
II68M
II85F

Figure S6

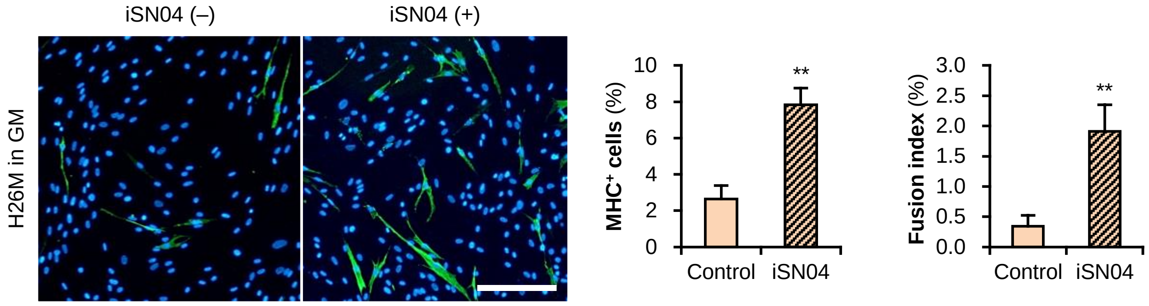
iSN04 (–)
iSN04 (+)
H26M in GM
MHC⁺ cells (%)
10
8
6
4
2
0
**
Control
iSN04
Fusion index (%)
3.0
2.5
2.0
1.5
1.0
0.5
0.0
**
Control
iSN04

Figure S7

A

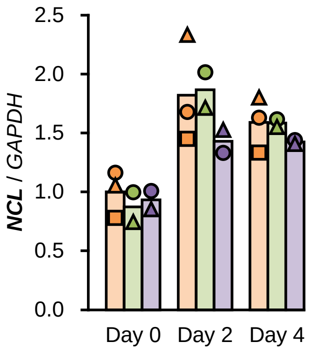

2.5
2.0
1.5
1.0
0.5
0.0
NCL / GAPDH
Day 0
Day 2
Day 4

B

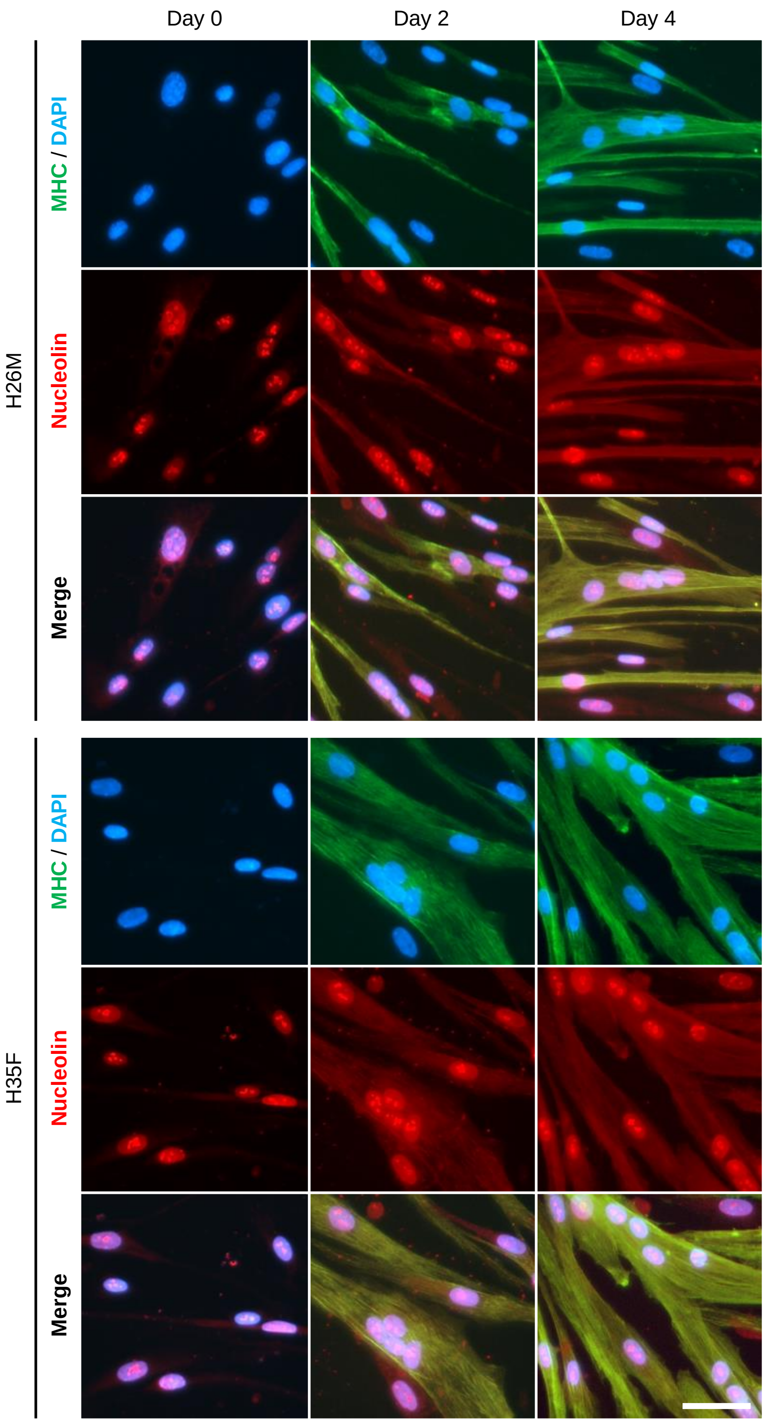

Day 0
Day 2
Day 4
H26M
MHC / DAPI
Nucleolin
Merge
H35F
MHC / DAPI
Nucleolin
Merge
